# Contraceptive decision-making and its association with contraceptive use among married adolescent girls in Niger

**DOI:** 10.1186/s12978-025-01962-x

**Published:** 2025-02-21

**Authors:** Jay G. Silverman, Shweta Tomar, Mohamad I. Brooks, Sani Aliou, Nicole E. Johns, Sneha Challa, Holly Baker Shakya, Sabrina C. Boyce, Anita Raj

**Affiliations:** 1 Center On Gender Equity and Health, School of Medicine, University of California San Diego, La Jolla, CA 92093 USA; 2 Pathfinder International, Watertown, MA 02472 USA; 3 Division of Community Health Sciences, School of Public Health, University of California, Berkeley, CA 94704 USA; 4 Newcomb Institute, Tulane University, New Orleans, LA 70118 USA; 5 Tulane School of Public Health and Hygiene, New Orleans, LA 70112 USA

**Keywords:** Contraceptive decision-making, Niger, Contraceptive use, Reproductive agency

## Abstract

**Objective:**

Niger has among the highest rates of child marriage and lowest rates of modern contraceptive use in the world. This study analyzes the association between contraceptive decision-making and contraceptive use among married adolescent girls in rural Niger, including multiple assessments of decision-making and consideration of overt vs. covert contraceptive use.

**Methods:**

We analyzed cross-sectional survey data collected from married adolescent females (n = 823) participating in the third round of data collection (October–November 2019) for the cluster-randomized controlled trial of a family planning intervention study. Contraceptive decision-making measures assessed participants’ (a) participation in contraceptive decision-making, (b) final say in decision-making in case of spousal disagreement, and (c) satisfaction with participation in decision-making. Outcomes include contraceptive use ever categorized based on whether use was overt (with husband’s knowledge) or covert (without husband’s knowledge). Adjusted multinomial logistic regression models were used to test the associations between each decision-making item and each type of contraceptive use.

**Results:**

Over half of participants reported ever using a contraceptive (59%) and that their husbands were the sole decision-makers regarding contraceptive use (60%). Adolescents’ participation in decision-making was negatively associated with overt contraceptive use (ARRR = 0.41; 95%CI = 0.19–0.91) and positively associated with covert contraceptive use (ARRR = 8.76; 95%CI = 2.45–31.30). Women reporting joint decision-making were more likely to report covert use vs. no use (ARRR = 3.20; 95%CI = 1.14–8.99). Women having final say in contraceptive decision-making in case of disagreements were more likely to report covert contraceptive use over no use (ARRR = 9.14; 95%CI = 3.17–26.40). Women’s satisfaction with decision-making was positively associated with contraceptive use ever (AOR = 2.72; 95%CI = 1.80–4.16), and overt (ARRR = 2.68; 95%CI = 1.75–4.01) and covert contraceptive use (ARRR = 10.9; 95%CI = 2.16–54.80).

**Conclusion:**

Male control over decision-making and female satisfaction with decision-making are associated with greater contraceptive use. Findings indicate that women’s control over decision-making, and its relation to contraceptive use, is complex and requires more nuanced understanding for married adolescents.

## Plain language summary

Niger is a country with one of the highest prevalence of child marriage in the world, with three in four girls marrying before the age of 18 years. Use of modern contraceptives remains low in this context with little known about the role of married adolescents in decisions regarding such use, or their control over or satisfaction with these decisions. In this study, we analyze data from a large representative sample of married adolescent girls in the Dosso region of Niger to access their participation in contraceptive decision-making, whether they or their husbands have the final say over such decisions in cases of disagreement, and how satisfied they are with the level of control they have over these decisions. In addition to describing agency regarding contraceptive use, we also assessed whether these aspects of decision-making are related to married adolescent girls’ reports of contraceptive use, and whether this use was with or without the knowledge of their husband. The results show that girls who participated in contraceptive decision-making alone or with their husbands, who had the final say in decision-making in cases of disagreement, and who reported being satisfied with their level of control over these decisions were more likely to use a contraceptive than others, but to do so without their husbands’ knowledge. The findings highlight the complexity of married adolescent girls’ reproductive autonomy in this context and indicate that measuring multiple aspects of contraceptive decision-making may advance a more nuanced understanding of their agency regarding these critical decisions.

## Background

Contraceptive use reduces maternal and infant mortality and supports advancement of women’s status and positioning by allowing women to choose whether and when to bear children [1, 2]. Marriage in adolescence can be a barrier to contraceptive use, as it often occurs in contexts where there is an expectation of early childbearing in marriage and less support for contraceptive use [3, 4]. Further, adolescent motherhood can compromise educational and economic opportunities for females [5, 6]. While adolescent marriage and childbearing has declined over the past few decades, corresponding with increases in contraceptive use, progress on these issues has slowed [4, 7], and a few countries, such as Niger, continue to see ongoing high rates of marriage and childbearing among adolescent girls [3, 8].

Every three in four girls in Niger is married before the age of 18 years; this is the highest prevalence of child marriage in the world [9]. Less than 12% of married girls in Niger use contraception, though more than one in five married adolescent girls in the country reported unmet need for birth spacing [10]. Higher wealth and education as well as urban residence were also associated with contraceptive use in Niger [10]. These findings suggest that at least some married adolescents in Niger want to use contraception, many of whom are contending with unmet need, and this unmet need may be higher for more socially and economically vulnerable girls. Less clear from these data is whether married girls have control over contraceptive decision-making in this context.

A review of the literature on contraceptive decision-making documents that female engagement in contraceptive decision-making with their male partner is a strong predictor of contraceptive use across national contexts [11], and a recent study focused on adolescent contraceptive decision-making in sub-Saharan Africa yields similar findings [12]. This latter study also found that among assessed nations, prevalence of requesting a partner use a condom and engagement in reproductive decision-making was among the lowest in Niger [12]. Unfortunately, the data from Niger across these studies is from five to ten years ago and may be less reflective of realities on the ground today, particularly in light of substantial commitments to family planning made in the country over the past decade [13]. Further, findings on contraceptive decision-making are largely limited to a question on female versus male versus joint decision-making on contraceptive use, but do not indicate women’s satisfaction with their level of control over decision-making or whether they would be the final decision-maker if there is not partner agreement. These are key aspects of whether decision-making is an act of reproductive agency for women, the purported goal of measuring this construct [11, 14, 15].

This study analyzes contraceptive decision-making and contraceptive use among married adolescent girls in rural areas of the Dosso Region, Niger, a context that retains social norms and expectations of adolescent marriage and childbearing as well as higher parity [16, 17]. Given that more traditional male partners may be unsupportive of contraceptive use in this context [16], especially for those adolescent wives who marry at very young ages [18], our contraceptive use outcome variable distinguishes between overt and covert use (i.e., use with and without knowledge of the partner). Moreover, covert contraceptive use has been documented in this population [19]. We also include a novel measure of contraceptive decision-making created for this study, which assesses women’s engagement in and satisfaction with decision-making, as well as her positioning as final decision-maker. This work offers insight into the needs of married adolescents in Niger and their reproductive agency [18, 20].

## Methods

### Study design

The present study used cross-sectional data from the third round of data collection (40 months after the baseline) in October–November 2019 taken from the cluster-randomized controlled trial of Reaching Married Adolescents, an intervention aimed at expanding access to modern contraceptive methods among married adolescents in Dosso, Niger. The trial used a four-arm (three intervention arms and one control arm) design and included three rounds of data collection (baseline, 24 months follow-up, and 40 months follow-up).

We used a two-stage sampling design, with stage one including a random selection of 16 villages from each of the three selected districts in Dosso: Dosso, Doutchi, and Loga. The eligibility criteria for the villages included: (1) having at least 1000 inhabitants and (2) village residents speaking either Zarma or Hausa language. Due to the logistical challenges of implementing all three intervention approaches in each district, we randomly assigned the three districts to an intervention arm, with control villages selected from within each district (12 intervention and four control villages from each district). The second stage of sampling involved listing all eligible married adolescent girls in the village, based on information provided by the village head, followed by a random selection of 25 girls and their husbands for participation in the study.

The eligibility criteria for adolescent girls included: (1) between 13 and 19 years of age at baseline, (2) married, (3) can speak Hausa or Zarma, (4) not having plans to move away in 18 months after the baseline study or plans to travel for more than six months during that time, (5) not currently sterilized, and (6) willing and able to provide informed consent. Further details of the research design can be found elsewhere [21]. A total of N = 1072 married adolescents completed the interview during baseline, out of which 823 adolescents completed the interview at the 40-month follow-up (retention rate = 77%). The sample for the current study included all the participants who completed the interview during the 40-month follow-up (N = 823).

Trained gender-matched, and French- and Hausa- or Zarma-speaking research assistants (RAs) conducted all interviews. The RAs approached the selected households, confirmed the eligibility of the selected participant, and obtained verbal consent from the household head and the study participant before starting the interviews. We replaced ineligible households and individuals who refused to participate in the study with a randomly selected household from the initial list. RAs conducted the surveys with women in a private location using an electronic tablet for data collection. Subsequent to data collection, staff then thanked the participants for their time. Participant privacy and confidentiality were maintained through separate encrypted file storage of any personally identifiable information collected, daily backup of survey data, and sharing/analysis of only de-identified data.

Due to the low literacy rate, participants provided verbal informed consent before survey participation. The Research Ethics Board of the Niger Ministry of Health and the University of California San Diego Institutional Review Board reviewed and approved the study protocols.

### Measures

The primary study outcomes included overall contraceptive use and contraceptive use type. The overall contraceptive use variable was categorized as 'ever' vs. 'never' based on the participants' response to the question- "Have you ever done anything or used any method to space or delay pregnancy." The participants who used contraceptives were asked if their husbands knew that they had ever done something or used a contraceptive method to space or delay pregnancy. Participants' response to this question was used to capture the contraceptive use type variable, which was categorized as—(1) no use, i.e., never used a modern contraceptive, (2) overt use, i.e., used a modern contraceptive ever, and husband knew about participant's modern contraceptive use, and (3) covert use, i.e., used a modern contraceptive ever but husband did not know about participant's modern contraceptive use.

The primary predictors included three variables measuring three aspects of contraceptive decision making: participation in decision-making, the final say in decision-making, and satisfaction with participation with decision-making. We asked participants, “Who usually makes decisions about contraceptive use?" Responses were "Husband alone," "Wife alone," or "Wife with husband." We then asked, "In case of disagreement, who makes the final decision about contraceptive use?". Responses were "Husband" or "Wife." We then asked participants, "Are you satisfied with the level of control you have over decisions about contraceptive use?" Responses to this item were "Never", "Rarely", "Usually," and "Always."

Covariates used in the models included participants' current age, age at marriage, parity, the age difference between husband and wife, education level, type of marriage, and household assets (a measure of wealth). The study arm and district were included as covariates to account for differences due to the study design. We captured participants' current age, their age at marriage, and the age difference between husband and wife as continuous variables in completed years. We measured parity as participants' total number of births (continuous variable). We defined participants' education as no modern schooling, only Quranic education, and no schooling. We categorized type of marriage as monogamous (one wife) or polygamous (two or more wives). The household asset score was calculated as the sum of items owned by the household, which ranged from 0 to 6. Study arm categories were intervention and control.

### Analysis

We used descriptive statistics, including proportions and means, to describe the sample, and we used t-tests, ANOVAs, and chi-squared tests to examine differences in demographics by contraceptive use outcome. Logistic regression models adjusted for covariates (demographics, treatment condition, and district) were used to test the association (adjusted odds ratio [AOR] and 95% CI) of decision-making variables with overall contraceptive use. Similarly, multinomial logistic regression models adjusted for covariates were used to test the association (adjusted relative risk ratio [ARRR] and 95% CI) of decision-making variables with type of contraceptive use. All the regression models included the treatment arm and district to adjust for any contextual difference due to the study design. However, we did not interpret the association of outcomes with these variables because these are relevant to the purpose of this analysis; impact of the trial by study arm is reported elsewhere [22]. We included the factors associated with the nonresponse rate at the 40-month follow-up round of data collection to address bias due to loss to follow-up. These factors included parity, the age difference between husband and wife, and asset score. We calculated the Generalized Variance Inflation Factor (GVIF) to test multicollinearity between the independent variables and found no multicollinearity at a GVIF cutoff level of 5. We also ran the Hausman-McFadden test to assess whether the assumption of Independence of Irrelevant Alternatives (IIA) was satisfied for our models. According to the IIA assumption, the odds ratio between two categories (of outcome variable) in a multinomial logistic regression should not depend on any other category. None of our models violated the IIA assumption. Data were analyzed using RStudio version 2021.9.0.351.

## Results

The final study sample included 823 married adolescents and young women. The mean age of the participants was 20.4 years, and their mean age at marriage was 14.1 years (Table 1). On average, the adolescent and young women were 8.3 years younger than their husbands and had an average of two children. Almost half (47.5%) of the participants reported not attending any modern or Quranic school. The majority (86.9%) of women reported being in a monogamous marriage. All the covariates were associated with both outcomes at p-values < 0.05 except age at marriage, which was not associated with contraceptive use ever, and asset score, which was not associated with either outcome.

**Table 1:** Sample demographics by outcomes

Demographic	Full sample	FP use ever			FP use type			
	N = 823	Never 41.4% (n = 340)	Ever 58.7% (n = 483)	p-value	No use 41.4% (n = 340)	Overt use 55.3% (n = 454)	Covert use 3.3% (n = 27)	p-value
	%/mean (n/sd)	%/mean (n/sd)	%/mean (n/sd)		%/mean (n/sd)	%/mean (n/sd)	%/mean (n/sd)	
Age	20.4 (1.5)	20.2 (1.6)	20.5 (1.5)	0.02	20.2 (1.6)	20.5 (1.5)	20.7 (1.2)	0.04
Age at marriage	14.1 (1.9)	14.2 (1.9)	14.1 (1.8)	0.29	14.2 (1.9)	14.0 (1.8)	14.9 (1.5)	0.03
Age difference between husband and wife	8.3 (5.0)	7.7 (4.7)	8.6 (5.2)	0.01	7.7 (4.7)	8.6 (5.2)	8.6 (5.5)	0.03
Parity	2.1 (1.3)	1.8 (1.4)	2.3 (1.7)	< 0.01	1.8 (1.4)	2.3 (1.1)	2.4 (1.7)	< 0.01
*Education*								
Any modern	36.1 (294)	30.3 (102)	40.3 (192)	0.01	30.3 (102)	41.1 (184)	25.9 (7)	0.02
Quranic only	16.3 (133)	16.6 (56)	16.1 (77)		16.6 (56)	15.6 (70)	22.2 (6)	
No education	47.5 (387)	53.1 (179)	43.6 (179)		53.1 (179)	43.3 (194)	51.6 (14)	
*Type of marriage*								
Monogamous	86.9 (695)	90.6 (300)	84.2 (395)	0.01	90.6 (300)	85.5 (377)	65.4 (17)	< 0.01
Polygamous	13.1 (387)	9.4 (31)	15.8 (74)		9.4 (31)	14.5 (64)	34.6 (9)	
Asset score	2.0 (1.2)	2.1 (1.1)	2.0 (1.2)	0.23	2.1 (1.1)	1.9 (1.2)	2.2 (1.3)	0.23
*Study arm*								
Treatment	76.1 (626)	68.5 (233)	18.6 (90)	< 0.01	68.5 (233)	81.7 (371)	74.0 (20)	< 0.01
Control	23.9 (197)	31.5 (107)	81.4 (393)		31.5 (107)	18.3 (83)	26.0 (7)	
*District*								
Dosso	33.6 (277)	34.1 (116)	33.3 (161)	< 0.01	34.1 (116)	33.5 (157)	33.3 (9)	0.02
Doutchi	31.8 (262)	25.9 (88)	36.0 (174)		25.9 (88)	36.1 (164)	29.6 (8)	
Loga	34.5 (284)	40.0 (136)	30.6 (148)		40.0 (136)	30.4 (138)	37.0 (10)	
*Who makes decision about FP use*								
Husband alone	60.0 (484)	59.1 (194)	60.5 (290)	0.76	59.1 (194)	62.7 (283)	22.2 (6)	< 0.01
Wife alone	4.8 (39)	5.5 (18)	4.4 (21)		5.5 (18)	2.9 (13)	29.6 (8)	
Wife with husband	35.2 (284)	35.4 (116)	35.1 (168)		35.4 (116)	34.4 (155)	48.2 (13)	
*Who makes decision in case of disagreement*								
Husband	93.7 (747)	94.3 (307)	92.6 (440)	0.16	94.3 (307)	94.2 (422)	65.4 (17)	< 0.01
Wife	6.3 (50)	4.7 (15)	7.4 (35)		4.7 (15)	5.8 (26)	34.6 (9)	
*Satisfaction with level of control over decision*								
Never satisfied	20.3 (161)	30.9 (97)	13.3 (64)	< 0.01	30.9 (97)	13.7 (62)	7.4 (2)	< 0.001
Rarely satisfied	11.2 (89)	15.3 (48)	8.5 (41)		15.3 (48)	8.2 (37)	14.8 (4)	
Usually satisfied	47.4 (376)	40.5 (127)	51.9 (249)		40.5 (127)	52.4 (237)	40.7 (11)	
Always satisfied	21.2 (168)	13.4 (42)	26.3 (126)		13.4 (42)	25.7 (116)	37.0 (10)	

The primary predictors of the study reflected that the husbands had more power over contraceptive decision-making for most participants. The majority (60.0%) of the women reported that their husband alone makes decisions about contraceptive use. The vast majority of women (93.7%) reported that their husbands make the decisions about contraceptive use in case of disagreement. Regarding satisfaction with their current level of decision-making control, almost half (47.4%) reported that they are usually satisfied with an additional 21.2% reporting that they are always satisfied with the level of control they have over contraceptive decision making. Contraceptive use (ever) was reported by more than half (58.7%) of participants, with 55.3% reporting overt use and 3.3% reporting covert contraceptive use.

Women’s participation in contraceptive decision-making (Table 2) was not associated with overall contraceptive use, but was negatively associated with overt contraceptive use and positively associated with covert contraceptive use. Specifically, those who made contraceptive decisions themselves (alone) were less likely to report overt use vs. no use (ARRR = 0.41; 95% CI = 0.19–0.91), more likely to report covert use vs. no use (ARRR = 8.76; 95% CI = 2.45–31.30), and more likely to report covert use vs. overt use (ARRR = 21.1; 95% CI = 5.76–77.60) compared to women whose husbands made contraceptive decisions alone. Women who reported joint contraceptive decision-making with their husbands were also more likely to report covert use vs. no use (ARRR = 3.20; 95% CI = 1.14–8.99) and more likely to use covert use vs. overt use (ARRR = 3.98; 95% CI = 1.43–11.10) than women whose husbands made all contraceptive decisions alone. Joint decision-making was not associated with overall or overt contraceptive use.

**Table 2:** Modeling logistic and multinomial regression models with participation in FP decision making as predictor

Predictor	FP use ever	FP use type		
	Ever vs never used	Overt vs no use	Covert vs no use	Covert vs overt use
	AOR (95% CI)^1,3^	ARRR (95% CI)^2,3^	ARRR (95% CI)^2,3^	ARRR (95% CI)^2,3^
*Participation in FP decision making*				
Husband alone	Ref.	Ref.	Ref.	Ref.
Wife alone	0.62 (0.30, 1.27)	0.41* (0.19, 0.91)	8.76*** (2.45, 31.30)	21.1*** (5.76, 77.60)
Wife with husband	0.86 (0.62, 1.19)	0.80 (0.60, 1.14)	3.20* (1.14, 8.99)	3.98** (1.43, 11.10)
Age	1.03 (0.92, 1,16)	1.03 (0.91, 1.14)	1.12 (0.78, 1.59)	1.09 (0.76, 1.54)
Age at marriage	1.02 (0.93, 1.12)	1.01 (0.90, 1.08)	1.24 (0.96, 1.60)	1.23 (0.96, 1.59)
Parity	1.39*** (1.21, 1.63)	1.39* (1.19, 1.60)	1.38* (1.01, 1.80)	0.99 (0.96, 1.59)
Age difference between husband and wife	1.01 (0.97, 1.05)	1.01 (0.97, 1.05)	0.95 (0.86, 1.05)	0.94 (0.85, 1.04)
*Education*				
Any modern	Ref.	Ref.	Ref.	Ref.
Quranic only	0.64^ (0.40, 1.02)	0.60* (0.37, 0.96)	1.56 (0.41, 6.01)	2.62 (0.69, 9.92)
No Education	0.68* (0.48, 0.96)	0.65* (0.46, 0.93)	1.25 (0.45, 3.53)	1.92 (0.69, 5.35)
*Type of marriage*				
Monogamous	Ref.	Ref.	Ref.	Ref.
Polygamous	1.65^ (0.96, 2.90)	1.47 (0.84, 2.60)	5.66** (1.78, 18.00)	3.84** (1.25, 11.8)
Asset score	0.93 (0.81, 1.06)	0.93 (0.81, 1.06)	0.98 (0.68, 0.90)	1.05 (0.73, 1.52)
*Study arm*				
Control	Ref.	Ref.	Ref.	Ref.
Treatment	2.17*** (1.52, 3.12)	2.24*** (1.56, 3.23)	1.31 (0.49, 3.53)	0.58 (0.22, 1.58)
*District*				
Dosso	Ref.	Ref.	Ref.	Ref.
Doutchi	1.54* (1.03, 2.32)	1.54* (1.02, 2.33)	1.08 (0.33, 3.54)	0.70 (0.22, 2.27)
Loga	0.67* (0.46, 0.98)	0.67* (0.45–0.97)	0.63 (0.22, 1.83)	0.95 (0.33, 2.74)

^1^AOR = Adjusted Odds Ratio, CI = Confidence Interval

^2^ARRR = Adjusted Relative Risk Ratio, CI = Confidence Interval

^3^Models adjusted for all demographics, study arm and district variables presented in the table

^ p-value < 0.1, * p-value < 0.05, ** p-value < 0.01, *** p-value < 0.001

Women having final say in contraceptive decision-making in cases of spousal disagreement (Table 3) was associated with covert contraceptive use vs. no use (ARRR = 9.14; 95% CI = 3.17–26.40) and covert use vs. no use (ARRR = 7.50; 2.77–20.3) but not with overall or overt contraceptive use.

**Table 3:** Modeling logistic and multinomial regression models with final say in FP decision making as predictor

Predictor	FP use ever	FP Use type		
	Ever vs never used	Overt vs no use	Covert vs no use	Covert vs overt use
	AOR (95% CI)^1,3^	ARRR (95% CI)^2,3^	ARRR (95% CI)^2,3^	ARRR (95% CI)^2,3^
*Final say in FP decision making*				
Husband	Ref.	Ref.	Ref.	Ref.
Wife	1.55 (0.81, 3.09)	1.22 (0.61, 2.45)	9.14*** (3.17, 26.40)	7.5*** (2.77, 20.3)
Age	1.04 (0.81, 3.09)	1.04 (0.92, 1.17)	1.06 (0.73, 1.54)	1.02 (0.71, 1.48)
Age at marriage	1.00 (0.91, 1.10)	0.99 (0.90, 1.09)	1.28^ (0.99, 1.67)	1.3** (1.00, 1.68)
Parity	1.35*** (1.16, 1.57)	1.33*** (1.14, 1.55)	1.64** (1.20, 2.23)	1.23 (0.92, 1.65)
Age difference between husband and wife	1.01 (0.97, 1.05)	1.01 (0.97–1.05)	0.94 (0.86, 1.04)	0.93 (0.85, 1.02)
*Education*				
Any modern	Ref.	Ref.	Ref.	Ref.
Quranic only	0.64^ (0.40, 1.02)	0.60* (0.38, 0.97)	1.63 (0.41, 6.33)	2.68 (0.70, 10.3)
No Education	0.67* (0.47, 0.95)	0.65* (0.46, 0.93)	1.25 (0.44, 3.54)	1.9 (0.68, 5.32)
*Type of marriage*				
Monogamous	Ref.	Ref.	Ref.	Ref.
Polygamous	1.62^ (0.94, 2.86)	1.46 (0.83, 2.58)	6.33** (1.96, 20.40)	4.33** (1.40, 13.3)
Asset score	0.91 (0.79, 1.04)	0.90 (0.79–1.03)	0.99 (0.67, 1.45)	1.09 (0.75, 1.59)
*Study arm*				
Control	Ref.	Ref.	Ref.	Ref.
Treatment	2.17*** (1.52, 3.11)	2.24*** (1.56, 3.22)	1.36 (0.50, 3.69)	0.61 (0.22, 1.64)
*District*				
Dosso	Ref.	Ref.	Ref.	Ref.
Doutchi	1.59* (1.06, 2.38)	1.59* (1.06, 2.38)	1.11 (0.34, 3.61)	0.7 (0.22, 2.22)
Loga	0.70^ (0.48, 1.02)	0.70^ (0.48, 1.02)	0.67 (0.23, 1.90)	0.95 (0.34, 2.68)

^1^AOR = Adjusted Odds Ratio, CI = Confidence Interval

^2^ARRR = Adjusted Relative Risk Ratio, CI = Confidence Interval

^3^Models adjusted for all demographics, study arm and district variables presented in the table

^ p-value < 0.1, * p-value < 0.05, ** p-value < 0.01, *** p-value < 0.001

Women’s satisfaction with contraceptive decision-making control (Table 4) was positively associated with both forms of contraceptive use. Compared to women who reported never being satisfied with their level of control over contraceptive decisions, those who were usually satisfied were more likely to report contraceptive use ever (AOR = 2.72; 95% CI = 1.80–4.16) and overt contraceptive use (ARRR = 2.68; 95% CI = 1.75–4.01) vs no use. Similarly, women who were always satisfied with their level of control over contraceptive decision-making were more likely to report both covert (ARRR = 10.9; 95% CI = 2.16–54.80) and overt contraceptive use (ARRR = 4.15; 95% CI = 2.47–6.95) vs no use. No association was observed between women’s satisfaction level and covert contraceptive use over no contraceptive use.

**Table 4:** Modeling logistic and multinomial regression models with satisfaction with FP decision making control as predictor

Predictor	FP use ever	FP Use type		
	Ever vs never used	Overt vs no use	Covert vs no use	Covert vs overt use
	AOR (95% CI)^1,3^	ARRR (95% CI)^2,3^	ARRR (95% CI)^2,3^	ARRR (95% CI)^2,3^
*Satisfaction with FP decision making control*				
Never satisfied	Ref.	Ref.	Ref.	Ref.
Rarely satisfied	1.30 (0.74, 2.29)	1.20 (0.67, 2.15)	4.52 (0.76, 27.00)	3.76 (0.62, 22.70)
Usually satisfied	2.72*** (1.80, 4.16)	2.68*** (1.75, 4.09)	3.56 (0.74, 17.30)	1.33 (0.27, 6.47)
Always satisfied	4.35*** (2.63, 7.32)	4.15*** (2.47, 6.95)	10.9*** (2.16, 54.80)	2.62 (0.53, 13.10)
Age	1.02 (0.91, 1.15)	1.02 (0.90, 1.15)	1.06 (0.75, 1.51)	1.04 (0.74, 1.47)
Age at marriage	1.01 (0.91, 1.11)	0.99 (0.90, 1.10)	1.31* (1.01, 1.70)	1.32** (1.02, 1.70)
Parity	1.38*** (1.19, 1.62)	1.37*** (1.17, 1.60)	1.60** (1.17, 2.20)	1.17 (0.87, 1.57)
Age difference between husband and wife	1.00 (0.96, 1.04)	1.00 (0.96, 1.04)	0.94 (0.86, 1.04)	0.94 (0.86, 1.03)
*Education*				
Any modern	Ref.	Ref.	Ref.	Ref.
Quranic only	0.69 (0.42, 1.13)	0.66 (0.40, 1.08)	1.49 (0.40, 5.60)	2.27 (0.62, 8.32)
No education	0.71^ (0.49, 1.02)	0.69* (0.48, 1.00)	1.31 (0.47, 3.64)	1.90 (0.70, 5.14)
*Type of marriage*				
Monogamous	Ref.	Ref.	Ref.	Ref.
Polygamous	1.91* (1.07, 3.49)	1.72^ (0.94, 3.12)	7.66*** (2.36, 24.9)	4.46** (1.47, 13.6)
Asset score	0.93 (0.81, 1.07)	0.93 (0.81, 1.07)	1.03 (0.71, 1.50)	1.11 (0.77, 1.60)
*Study arm*				
Control	Ref.	Ref.	Ref.	Ref.
Treatment	2.04*** (1.41, 2.96)	2.10*** (1.44, 3.05)	1.41 (0.53, 3.75)	0.67 (0.25, 1.77)
*District*				
Dosso	Ref.	Ref.	Ref.	Ref.
Doutchi	1.53* (1.01, 2.34)	1.55* (1.02, 2.37)	1.03 (0.33, 3.23)	0.66 (0.22, 2.02)
Loga	0.82 (0.55, 1.22)	0.81 (0.55, 1.21)	0.84 (0.30, 2.33)	1.03 (0.38, 2.81)

^1^AOR = Adjusted Odds Ratio, CI = Confidence Interval

^2^ARRR = Adjusted Relative Risk Ratio, CI = Confidence Interval

^3^Models adjusted for all demographics, study arm and district variables presented in the table

^ p-value < 0.1, * p-value < 0.05, ** p-value < 0.01, *** p-value < 0.001

## Discussion

The study contributes novel findings on multiple aspects of contraceptive decision-making (participation in, control over and satisfaction with level of control over decision-making) and their associations with contraceptive use (overall, overt use and covert use) among young married women in rural Niger. The findings indicate that, in this context, young married women have low levels of both participation and control over contraceptive decision-making but were usually or always satisfied with their level of control in these situations. Furthermore, associations between decision-making and contraceptive use differed based on the different aspects of decision-making and whether contraceptive use was overt or covert.

Specifically, approximately two-thirds of participating married adolescents in Niger reported having no role in decisions regarding their contraceptive use. Although these findings appear to be inconsistent with other studies in the sub-Saharan Africa context where joint decision-making is most commonly reported [23, 24], the current sample is composed only of younger women who were married before age 18 years, and both younger age and early marriage have been observed to be associated with lower participation in such decisions [18, 25]. Notably, associations of decision-making with contraceptive use differed based on both aspect of decision-making and whether contraceptive use was overt or covert.

When contraceptive use was assessed with consideration of whether such use was overt or covert, use was not associated with level of young women’s participation in decisions regarding contraceptive use. This finding is inconsistent with prior research [11, 23, 26] indicating that joint contraceptive decision-making is positively associated with contraceptive use. As discussed above, the demographics of this sample, specifically the younger age and early marriage, make comparison of the current results with previous findings on contraceptive use and decision-making among older samples of women likely inappropriate.

Assessing covert contraceptive use vs. no use or overt, covert use was seen to be positively associated with young women’s participation in decisions regarding contraceptive use, whether alone or jointly with their husband, vs. their not participating in this decision-making. In contrast, overt use of contraception was less likely in situations where young women did not participate in this decision-making. This is consistent with earlier studies of this same population that found reproductive coercion (i.e., men’s behaviors that reduce female control over contraceptive use and pregnancy) was found to be positively associated with covert contraceptive use [19].

Young married women’s control of contraceptive decision-making in cases of spousal disagreement about use was rare (6%) but strongly associated with covert use and not associated with overt use. Although there is little research on control of contraceptive decisions in case of conflict, the earlier study discussed above on reproductive coercion and use also found this pattern of associations [19] which is not surprising given spousal conflict over contraceptive decisions is typically the context for male partner behaviors related to reproductive coercion (e.g., taking away or hiding contraceptive methods).

Importantly, both the findings regarding contraceptive decision-making participation and control in cases of spousal disagreement suggest that the standard assessments of both contraceptive decision-making, which excludes cases of disagreement, and of contraceptive use, which excludes consideration of whether such use if covert or overt, may be inadequate to provide a clear understanding of female control of contraceptive decisions and use, specifically female contraceptive choice (based on knowledge and critical consciousness of the existence of choice) and agency (ability to act on a choice). For example, although joint participation in contraceptive decision-making was reported by 35% of young married women, only 6% reported controlling this decision in cases of disagreement. Joint decision-making was not associated with contraceptive use in total, but was strongly associated with covert use. Women reporting that they alone participate in contraceptive decisions were not only more likely to report covert use, they were significantly less likely to report overt contraceptive use. Similarly, women’s control of contraceptive decisions in cases of spousal disagreement was associated with covert and not overt use.

In addition, findings from the current study support inclusion of women’s satisfaction with decision-making, and not just their decision-making involvement, in understanding agency and choice. Results indicate that young married women are more satisfied with their level of decision-making involvement when they are using contraceptives, whether overtly or covertly. This finding is consistent with a recent study of contraceptive decision-making that also included assessment of women’s satisfaction with their level of decision-making control conducted among married women in Nepal [27]. Here too, study results showed that women’s satisfaction with FP decision-making control were more likely to report contraceptive use ever and with both overt FP use and covert use, with the strength of the association being strongest for covert use [27]. Prior research with this same sample in Niger also found that those experiencing physical intimate partner violence and reproductive coercion are more likely to engage in covert use [19]. Taken together, these findings suggest that married adolescents who are contending with an abusive or controlling husband may find it worth going against the norms of both non-use of contraceptives and male control over contraceptive decision-making to control their own fertility and prevent childbirth.

Finally, these findings suggest that lack of participation or control over decisions, in this context, may not equate to young married women’s dissatisfaction with their relative role in this regard. Although 60% of participants reported not participating in contraceptive decisions and 94% reported that their husband’s choice prevails when there is a disagreement about contraceptive use, 69% reported being usually or always satisfied with their level of control in these situations. Further, as described above, high satisfaction was associated with increased likelihood of contraceptive use. In patriarchal contexts such as rural Niger, men pushing against traditional social norms, such as those against use of contraceptives, are likely to be subject to less backlash than women pushing against such norms, and this may be particularly true for adolescent married women who likely have even less power than other women. This is borne out by the current findings that women who participate in or control contraceptive decision-making are more likely to resort to covert use. These findings indicate support for increased efforts to engage men to support contraceptive use in contexts where men dominate related decisions. However, without changing the existing dynamic of male control over women’s fertility, are we supporting women’s reproductive choice and agency, or are we simply supporting women’s contraceptive use, regardless of her interests? If the latter, is this approach counter to the global aid and public health communities’ increasing focus on women’s empowerment and gender transformative change in the context of reproductive health?

A major limitation of this study is generalizability. The majority of married adolescent women (59%) reported contraceptive use ever, a prevalence much higher than that seen for the general population [9, 10]. Use of follow-up data from a family planning intervention study is likely responsible for this relatively high level of contraceptive use, and make the sample unique from the general population of women in Niger. Importantly, this larger number of contraceptive users provided sufficient power to conduct the analyses described. Another limitation is the lack of data on specific contraceptive methods used by participants. This information would have been valuable in understanding how method choice relates to covert use patterns, as some methods are more amenable to discreet use than others (e.g., injectable contraceptives versus oral contraceptives). Due to lack of this data, we were unable to examine whether women who reported joint or husband-led decision-making chose different methods than their agreed-upon method, which could explain covert use in case of joint decision-making. Additional limitations relate to reliance on self-report measures, potentially resulting in social desirability biases, and focus on current decision-making rather than ever decision-making, with use of ever use of contraceptives as outcomes.

## Conclusion

The current findings highlight the complexity of women’s decision-making control over family planning being a central indicator of women’s reproductive autonomy. They also indicate the potential value of a more comprehensive measure of contraceptive decision-making, inclusive of women’s control over final decision-making and satisfaction with decision-making involvement, to guide understanding of women’s reproductive agency, with the study of gender and social norms regarding contraceptive decision-making a likely critical component of such research.

## Data Availability

De-identified data and analysis code are available from the corresponding author upon reasonable request.

## Abbreviations

ARRR: Adjusted relative risk ratio
AOR: Adjusted odds ratio
CI: Confidence interval
RA: Research assistants
